# The Context of the Emergency Department as a Location for a Smoking Cessation Intervention—Process Evaluation Findings From the Cessation of Smoking Trial in the Emergency Department Trial

**DOI:** 10.1093/ntr/ntae223

**Published:** 2024-11-07

**Authors:** Caitlin Notley, Pippa Belderson, Emma Ward, Lucy V Clark, Allan Clark, Susan Stirling, Steve Parrott, Jinshuo Li, Timothy J Coats, Linda Bauld, Richard Holland, Sarah Gentry, Sanjay Agrawal, Benjamin M Bloom, Adrian Boyle, Alasdair Gray, M Geraint Morris, Ian Pope

**Affiliations:** Norwich Medical School, University of East Anglia, Norwich, UK; Norwich Medical School, University of East Anglia, Norwich, UK; Norwich Medical School, University of East Anglia, Norwich, UK; Norwich Clinical Trials Unit, Norwich Medical School, University of East Anglia, Norwich, UK; Norwich Clinical Trials Unit, Norwich Medical School, University of East Anglia, Norwich, UK; Norwich Clinical Trials Unit, Norwich Medical School, University of East Anglia, Norwich, UK; Department of Health Sciences, University of York, York, UK; Norwich Medical School, University of East Anglia, Norwich, UK; Department of Health Sciences, University of York, York, UK; Department of Cardiovascular Sciences, Leicester Medical School, University of Leicester, Leicester, UK; Usher Institute and SPECTRUM Consortium, College of Medicine, University of Edinburgh, Edinbugh, UK; Exeter Medical School, University of Exeter, Exeter, UK; Norwich Medical School, University of East Anglia, Norwich, UK; University Hospitals of Leicester NHS Trust, Leicester, UK; Royal London Hospital, Barts NHS Trust, London, UK; Addenbrookes Hospital, Cambridge University Hospitals Foundation Trust, Cambridge, UK; Royal Infirmary Edinburgh, NHS Lothian, Edinburgh, UK; Homerton University Hospital NHS Trust, London, UK; Norwich Medical School, University of East Anglia, Norwich, UK; Norfolk and Norwich University Hospital, Norwich, UK

## Abstract

**Introduction:**

Hospital emergency departments (ED) offer an opportunity to engage with large numbers of people who smoke to prompt cessation, although the acceptability of opportunistic intervention in this context has been questioned. This process evaluation study was embedded into the Cessation of Smoking Trial in the Emergency Department (COSTED) randomized controlled trial and sought to explore the context of intervention delivery within the ED.

**Aims and Methods:**

Qualitative interviews were conducted with participants and staff across six EDs participating in the COSTED randomized controlled trial. Interview data were thematically analyzed specifically exploring contextual influences. Data were triangulated with ethnographic observations.

**Results:**

In participant interviews (*N* = 34), it was acceptable overall to receive a brief opportunistic smoking cessation intervention in the ED. Contextual factors are impacted at a range of levels. At the micro level participant views and experiences combined with staff tailoring were important. Being given an e-cigarette starter kit by a “credible source” helped to legitimize vaping for smoking cessation and gave confidence in personal ability to switch away from tobacco. At the meso level, adaptations to intervention delivery were made in response to the context of the ED. Stop smoking advisors (*N* = 11) had to adapt and deliver the intervention flexibly depending on space and clinical need. At the macro level, hospital policies supportive of vaping legitimized the approach.

**Conclusions:**

Smoking cessation outcomes reported in the main trial across sites were very similar because of the high credibility, acceptability, and flexible approach to delivering the COSTED intervention in the ED.

**Implications:**

Attending a hospital ED is the right time and place to receive smoking cessation intervention, even for those not motivated to quit. People are willing to receive intervention, and clinical staff are willing to support intervention delivery. Despite challenges, overall the context is helpful in supporting people to switch away from tobacco. The intervention, with flexible and tailored implementation, is adaptable to different ED contexts. This suggests that wider implementation across NHS Trusts of the effective COSTED intervention is feasible and will ultimately support smoking cessation for people attending EDs, who may not otherwise have sought support.

## Introduction

Smoking tobacco is a key driver of health inequalities. The more disadvantaged someone is, the more likely they are to smoke tobacco,^1^ suffer from smoking-related diseases and die prematurely. Smoking prevalence is high amongst people attending hospital emergency departments (EDs),^2^ who also treat disproportionate numbers of people from lower socioeconomic groups.^3^ The ED presents an opportunistic setting to support people to quit smoking.^4,5^

The Cessation of Smoking Trial in the Emergency Department (COSTED)^6,7^ tested the real-world effectiveness of an ED-based brief intervention to support people to quit smoking. The intervention included brief advice, provision of an e-cigarette starter kit and referral to stop smoking services. The trial demonstrated that the intervention was effective at supporting long-term biochemically validated continuous smoking abstinence for people (motivated or unmotivated) attending a hospital ED.^7^

It is increasingly recognized that context fundamentally impacts intervention delivery, both within randomized controlled trials and real-world implementation.^8^ We define context as “any feature of the circumstances in which an intervention is conceived, developed, implemented, and evaluated.”^9^ The ED context represents a frenetic clinical environment where large numbers of patients are assessed, triaged, and treated. It has been assumed that this environment is not conducive to the implementation of behavioral interventions due to urgent clinical demands and staff burden.^10^ This suggests that careful consideration of the context for intervention delivery, its acceptability and appropriateness, from multiple perspectives, is key.^9^ Within the COSTED trial, this embedded process evaluation sought to explore intervention delivery and implementation, recognizing that RCTs can inform understanding of effectiveness, but other approaches are needed to inform “why things work.”^11^ To the best of our knowledge, this study is the first to fully explore smoking cessation intervention delivery in the ED context.

## Methods

A mixed methods qualitative process evaluation^12^ was undertaken to assess implementation of the intervention within the ED and to explore participant and staff views on the intervention, contextual variation, and the impact of context. Ethical approval for the study was granted (South Central—Oxford B Research Committee 21/SC/0288; Trial Registration number: NCT04854616) and the analysis and findings are reported according to COREQ guidelines.^13^

Detailed qualitative data were collected from participants and clinical staff by the experienced research team (CN, PB, and EW undertook observations and interviews).

Observations at the six EDs in which the interventions were being delivered were undertaken with detailed field notes taken. Interviews were undertaken with Advisors delivering the intervention from each site.

Trial participants were purposively selected for interviews across both intervention and control groups and across all sites following the final trial follow-up (6 months). The sample size was determined based on the likely “sufficiency” of data to explore the question of contextual influence on intervention delivery. Participants were invited to participate in in-depth interviews, for which they gave written consent. Interview guides, developed in consultation with patient and public (PPI) representatives, explored views and experiences of the intervention where received, to understand barriers and facilitators, and to assess patients’ perspectives. Interviews took place over the telephone or via video call, lasted between 11 and 75 minutes, and were audio recorded and transcribed verbatim.

As our focus for analysis was understanding the impact of context on intervention delivery, we devised a thematic coding frame to enable an analysis of contextual impacts at different levels, theoretically driven according to the social-ecological framework.^14^ This enabled us to consider influence at the microsystem (eg, interpersonal staff-patient interactions) and individual (eg, intraindividual participant thoughts and perspectives), mesosystem (eg, the micro-social context of the ED setting), and macrosystem levels (eg, policy). Inductive thematic analysis^15^ of interview data commenced using NVivo until “analytical sufficiency”^16^ was reached. This was supplemented with second-stage coding, organizing codes into levels of contextual influence. PB and EW undertook the thematic analysis, with CN verifying the analysis. Interpretation of codes was discussed and agreed upon by consensus. Findings are reported integrating observational and quantitative data to inform an understanding of the ED context for trial outcomes. Quotations are used as illustrations of the themes presented, with all participants fully anonymized.

## Results

Qualitative interviews captured the views of a range of ED-based Advisors involved in delivering the intervention including research nurses, ED health care assistants, and clinical research practitioners(*n* = 11) across all sites. Our purposive sampling of trial participants meant that we spoke to a broad range of people representing diverse characteristics (*N* = 34) (Table 1).

**Table 1.** Participant Interview Sample

	Trial participants (interview subsample *n* = 34)
*Site*	
Site 1	12
Site 2	6
Site 3	6
Site 4	3
Site 5	5
Site 6	2
*Sample group*	
Intervention—Quit	7
Intervention—Harm reduction (reduced cigarettes per day by at least 50%)	12
Intervention—No/ limited change	5
Usual Care	10
*Gender*	
Male	20
Female	14
*Age (years)*	
Mean (range)	44 (20–70)
20–29	6
30–39	8
40–49	5
50–59	10
≥60	5
*Ethnicity*	
Asian Bangladeshi	2
Asian Other	1
Black British	1
Black Caribbean	2
White British	23
White Eastern European	2
White Irish	2
White Other	1
*Employment status*	
Employed full-time	15
Employed part-time	4
Full-time carer (eg, of children or other family members)	1
Retired	2
Self-employed or freelance	6
Unable to work due to sickness or disability	4
Unemployed and looking for work	2

The six EDs were situated across England and Scotland (see table in Supplementary Appendix S1 for contextual notes).

The ED context was found to profoundly impact both intervention delivery and how participants received the intervention. Despite this, overall the effect of context did not significantly impact the primary outcome of biochemically verified smoking cessation by site (Tables 2 and 3).

**Table 2.** Smoking Abstinence at 6 Months (Biochemically Validated by CO Reading), By Site

	ITT population		Minimally adjusted			
Outcome	Control (*n* = 488)	Intervention (*n* = 484)	Relative risk (95% CI)	*p*-value	Difference in risk (95% CI)	*p*-value
Self-reported continuous smoking abstinence at 6 m, biochemically validated (CO reading ≤7), *n*(%)	20 (4.1%)	35 (7.2%)	1.76 (1.03, 3.01)	.042	0.031 (0.002, 0.060)	.035
**By site** ^*^:						
Site 1	10 (5.0%) (*n* = 201)	14 (7.0%) (*n* = 199)	1.41 (0.64,3.11)	.389	0.02 (−0.03, 0.07)	0.386
Site 2	3 (3.6%) (*n* = 84)	5 (6.0%) (*n* = 84)	1.67 (0.41, 6.75)	.474	0.02 (−0.04, 0.09)	0.468
Site 3	2 (3.8%) (*n* = 53)	6 (11.1%) (*n* = 54)	2.94 (0.62, 13.94)	.173	0.07 (−0.02, 0.17)	0.143
Site 4	3 (4.0%) (*n* = 76)	5 (6.8%) (*n* = 74)	1.71 (0.42, 6.91)	.450	0.03 (−0.04, 0.10)	0.445
Site 5	2 (4.0%) (*n* = 50)	3 (6.0%) (*n* = 50)	1.5 (0.26, 8.60)	.649	0.02 (−0.07, 0.11)	0.646
Site 6	0 (*n* = 24)	2 (8.7%) (*n* = 23)	NA		NA	

^*^Test for interaction: *p* = .9454.

**Table 3.** Self-reported Smoking Abstinence at 6 Months (Not Biochemically Validated), By Site

	ITT population		Minimally adjusted			
Outcome	Control (*n* = 488)	Intervention (*n* = 484)	Relative risk (95% CI)	*p*-value	Difference in risk (95% CI)	*p*-value
Self-reported smoking abstinence at 6m	64 (13.1%)	122 (25.2%)	1.92 (1.46, 2.53)	<.001	0.12 (0.07, 0.17)	<.001
By site^*^:						
Site 1	24(11.9%) (*n* = 201)	48 (24.1%) (*n* = 199)	2.02 (1.29, 3.17)	.002	0.12 (0.05, 0.20)	.001
Site 2	13(15.5%) (*n* = 84)	22 (26.2%) (*n* = 84)	1.69 (0.91, 3.13)	.094	0.11 (−0.01, 0.23)	.085
Site 3	9 (17.0%) (*n* = 53)	13 (24.1%) (*n* = 54)	1.42 (0.67, 3.03)	.369	0.07 (−0.08, 0.22)	.362
Site 4	8 (10.5%) (*n* = 76)	18 (24.3%) (*n* = 74)	2.31 (1.07, 4.99)	.033	0.14 (0.02, 0.26)	.024
Site 5	9 (18.0%) (*n* = 50)	16 (32.0%) (*n* = 50)	1.78 (0.87, 3.64)	.115	0.14 (−0.03, 0.31)	.101
Site 6	1 (4.2%) (*n* = 24)	5 (21.7%) (*n* = 23)	5.22 (0.66, 41.32)	.118	0.18 (−0.01, 0.36)	.065

^*^Test for interaction: *p* = .8191.

Qualitative data revealed contextual influences that influenced how staff delivered, and how participants received, the intervention at different levels.

### Microsystem Contextual Influences

At the individual and interpersonal level, specific factors, such as presenting condition, motivation to change behavior and previous experiences, impacted the way in which the intervention was “received.”

#### “Right Time, Right Place”

Although participants were attending the ED for urgent care, perhaps completely unrelated to tobacco smoking, most described being offered smoking cessation support at an opportune time, catching a “teachable moment” where they were amenable to considering changing their smoking behavior:

*“I don’t know whether it was just the right place, right time or the way that they said it. It was just, it was different. It made me think more about it*” [01-0248: female, age 22, intervention participant]

*“I was there with a health issue. And it was a case of well, if I’m in here getting one thing sorted, why not get something else sorted at the same time?”.* [06-0020: male, age 51, intervention participant]

For some, the intervention had just happened to catch them at a “turning point” when they were motivated to change:

*“I think I’ve always known that I shouldn’t be doing it and in the back of my mind I’ve thought that I need to get that sorted. Maybe it is a good time to approach the subject with people in a different way.”* [05-0209: female, age 52, intervention participant]

For others, motivation to quit smoking was less, but the shock of the acute medical situation requiring ED attendance prompted reflection and made the opportunity to consider cessation more acceptable:

*“I do think that people are feeling just perhaps more vulnerable when they’re sitting in A&E. They’re more aware of their own mortality, else they wouldn’t be there. So, there’s that kind of psychological aspect to it”* [03-0127: female, age 61, intervention participant]

#### Reassurance From a “Credible Source”

For many of the participants, having the intervention delivered by an advisor, who was deemed to be a clinical professional, gave it credibility, offering reassurance:

“*I was really unsure about the vape until I was in hospital. When they suggested it, I thought great, maybe it’s not as bad as everyone was suggesting it was. If someone in hospital is offering to put you on that, it’s got to be better than smoking”* [05-0117: female, age 41, intervention participant]

Specifically, perceiving the advisors to be “qualified medical professionals” seemed impactful to participants:

*“I was receiving it from qualified professionals…I think it was a very good opportunity that the doctor did inquire about smoking and stuff and asking would I like to be part of this study… I mean these people are qualified, that’s why I’m commenting here. I just think, I’ll take their word for it.* [02-0520: male, age 31, intervention participant]

#### Interpretation of the Intervention Manual and Relational Adaptations

Adaptability and tailoring the intervention manual to the participant and presenting condition were key. Advisors demonstrated the ability to respond sensitively to information and demeanor of individuals, and to personalize the intervention:

“*You have to be tactful with it, I think. Not preachy. You have to try to personalize it to them. But, and a lot of people wanted to talk a lot about why they were there and some of it wasn’t relevant, but they want to talk. But then if you can latch onto something that’s pertinent to them, that makes the rest a bit easier.”* [Advisor 01-06]

Critically, *listening* and responding appropriately to the individual was a major aspect of adapting the intervention delivery:

*“I probably tailor it as well. You have to learn, I think, to tailor the intervention to the person that you’re talking to rather than the script….You have to make that judgment and let people talk to you.”* [Advisor 01-02]

Despite these adaptations and “in the moment” tailoring, observation and interview data suggested good adherence and fidelity to the intervention manual overall. Specifically, observations revealed how staff responded to participants with rapport and empathy, being aware of possible stigma around smoking, and therefore talking “with” rather than “at” participants:

“*I think the listening and being open minded and adaptable…because everybody is different and try to get them to have a conversation with you rather than jamming information down the throat.”* [Advisor 04-02]

Observations gave confidence that advisors had a good level of understanding of smoking cessation, the health benefits of switching to vaping, and detailed technical knowledge about the specific device provided. This demonstrable knowledge ensured that key intervention components were delivered, and fidelity to the manual was high overall. However, some deviations in intervention delivery were noted:

“*I probably didn’t use the script as much. Just because I didn’t want to come across as though I was just reading to the participant. But having the bullet points, the facts and the FAQs was really helpful.”* [Advisor 05-03]

#### Advisor “Buy-In”

The personal view, training, and level of understanding of advisors were an immediate contextual factor that had a strong influence on intervention delivery. Generally, we observed extremely good commitment and “buy in” to the importance of the intervention. Feedback on the training provided was positive, as advisors viewed it as an opportunity to upskill and build knowledge. Several advisers spoke about how it had changed their perspective and understanding of smoking cessation:

“*I found it really, personally, really interesting information and when I was sat there doing it, my personal thoughts at the time were that every healthcare professional should get this training.”* [Advisor 06-02]

Individual advisors who themselves reported being an ex-smoker or vaper had a particular level of insight that was facilitative:

“*I used to socially smoke when I was like, my early 20s and I really pulled on that. That would really help build a rapport. Even though I haven’t smoked in such a long time. But if I then said, “oh, when I used to smoke, I know it’s very difficult.”*[Advisor 02-08]

Advisors had a variety of professional backgrounds, including research nurses, ED nurses, healthcare assistants, and peer support workers. Each brought a unique skill set and experience but received the standardized COSTED training package. Although different backgrounds played out in different ways, all managed to successfully recruit and there were no discernible across-site differences in smoking cessation outcomes. What seemed critical to the successful delivery of the intervention was the overall enthusiasm and “buy in” of advisors:

“*You can’t convert everyone, but there’s some you really wanted to get the buzz and take it on board and just catch some enthusiasm. And I felt that I was quite enthusiastic about it and you just want everybody to feel it…it was quite an inspiring thing to do really. You just hope that you’ve touched a few of the lives of the people you meet”* [Advisor: 01-08]

#### ED Staff Barriers and Facilitators

In staff interviews it was apparent that some other members of ED staff were resistant to the intervention approach, perhaps because they smoked themselves, or more commonly, did not see the intervention as being compatible with the ED setting:

*“Because we’ve got a few smokers ourselves, so it depends very much on whether or not they were a smoker or non-smoker…it was hard work trying to get them to engage… I think people and staff wouldn’t necessarily associate quitting smoking in an ED setting.”* [Advisor 06-02]

Advisors reported that they sometimes perceived being viewed as an obstruction:

*“Input from the ED staff was minimal. I think it was just another thing that they didn’t have time to do. “* [Advisor 04-02]

Conversely, individual medical staff with personal interests could be very supportive, which was highly facilitative to the research generally and intervention delivery specifically:

*“Some actively sought us out and said, ‘oh, I’ve got a smoker’ and stuff like that…you’d get some of the other doctors come out and say ‘I’ve got somebody for you’. It was completely mixed. We had a lot of staff who were very interested in what we were doing”* [Advisor 01-06]

### Mesosystem Contextual Influences

At the mesosystem level aspects of intervention delivery specifically within the setting of a hospital appeared to be highly important in terms of how the intervention was both delivered and received.

#### Legitimization of Vaping

Many participants talked about having tried vaping previously, unsuccessfully as part of a quit attempt, or experimentally, and not finding it enjoyable, satisfying, or effective as a smoking replacement. These previous experiences colored views and expectations, but the offer of the vape within the ED facilitated, for some, a re-think about considering vaping as a way of supporting smoking cessation.

Others had never tried vaping before, but had concerns about safety, perhaps due to media stories they had read. The offer of the vape within a medical setting legitimized it as an acceptable and “approved” method of smoking cessation:

*“I was really unsure about the vape until I was in hospital. When they suggested it, I thought great, maybe it’s not as bad as everyone was suggesting it was. If someone in hospital is offering to put you on that, it’s got to be better than smoking”* [05-0177: female, age 41, usual care]

For some, perceptions of vapers, or what might be perceived as a “vaping identity,” were negative. However, being offered a vape in the context of the ED legitimized the vape as a smoking cessation intervention:

*“It was my first-time in my life vaping literally, because I’ve always been so sceptical about it. Like I said, I’ve seen friends, I’ve seen people around it and I would just be that stupid thought like, “Yeah, you know, you’re a sissy, you’re not smoking.” You understand that? It was the mindset I had; due to the place I grew up in unfortunately”* [02-0520: male, age 31, intervention participant]

#### The Nature of the ED and Waiting Times

Lengthy ED waiting times seemed to play a positive role in facilitating acceptability. From the perspectives of participants, waiting acted as a positive incentive to take part in the study, since they were resigned to “passing the time”:

*“In A&E, I was stuck there so, I was there for a couple of hours as it was so, it probably increased my likelihood to actually enrol.”* [01-0841: male, age 28, intervention participant]

Advisors were able to turn the long waiting times around to positively intervene with patients:

*“When they were all queuing in there and they were fed up waiting, we’d say to them you’ve got a long wait, would you like to come and speak to us while waiting? It’ll help pass the time. That worked quite well.”* [Advisor 01-06]

Others were glad of the distraction from pain or worry:

*“I was kind of focused on like the pain in my knee and it kind of helped to distract me when I was there, because I could talk about this instead.”* [03-0065: female, age 28, intervention participant]

#### Working Within ED Clinical Care Pathways

A core concern of staff delivering the intervention was awareness of the ED context, where immediate medical care took precedence. This meant dealing with interruptions for clinical care and the possibility of participants moving on or being “lost”:

*“We did lose a couple….because once they are called to the doctors and they’ve had the treatment, you won’t get them later… they’ve had enough.”* [Advisor 01-06]

For some advisors, it was also a challenge to deliver the intervention, as patients could be anxious about missing clinical interventions:

*“I felt like for some patients they were the ones rushing me to finish because perhaps they were anxious that they were missing something …some of those were really rushed, so I had to sort of like cherry pick what was gonna be most important.”* [Advisor 04-02]

However, despite challenges, advisers generally found patients accepting of interruptions and adaptations to fit the intervention around their clinical care. Within the sample, we recorded instances of the intervention being delivered before, during, and after clinical investigations, and even immediately following patient discharge, demonstrating high levels of flexibility in the intervention delivery:

*“You have to accept that you need to be flexible with where you’re going to do it. The needs of the department always take precedence. You adapt to suit that… People are quite accepting of starting here but moving to here to finish things*.” [Advisor 05-09]

#### Availability of Appropriate Space for Delivery

The availability of space to deliver the intervention varied across sites. Our observations revealed flexibility in the use of locations, including the waiting room, trolley space, consulting rooms, and outside (where a participant wanted to try the vape). Advisors demonstrated a pragmatic approach in where they delivered the intervention, clearly making adaptations as necessary.

However, distraction and noise in the ED environment were reported as a barrier to intervention delivery, although this seemed more of a staff concern relating to privacy than a patient concern:

*“A lot of the time, the patients were happy to just sit where they were and do the intervention although that impacted how much they would want to open up.”* [Advisor 04-02]

Despite being less than ideal, pressures on space for intervention delivery did not appear to act negatively, overall, on participant experience:

“*Delivering the intervention was never a problem….Most of the time it was delivered in the waiting room. That was fine. Delivered in majors, even better. They’ve got their own cubicle.”* [Advisor 03-02]

### Macrosystem Contextual Influences

In consideration of the wider policy and environmental context, hospital vaping policies and practices, which varied between sites, were extremely important to legitimizing the intervention approach. From the participant’s perspective, site policies were noticed. At some sites, smoking was banned on the premises but vaping was permitted. Particularly in contexts where “smoke-free” signage was combined with “vape friendly” site policies, the environment was felt to further legitimize and support vaping as part of a smoking quit attempt:

*“It would influence me because even if I wasn’t leaving to smoke, I’d think hold on, smoking is not allowed, but they think vaping allowed. This is in a hospital...”* [02-0520: male, age 31, intervention participant]

For staff delivering the intervention, sites that had a “vape friendly policy” gave advisors confidence to suggest trying the vape there and then, on site:

“*I think it was quite helpful that people were absolutely gasping for a cigarette, so we could say to them well you can take this outside or if we take you outside to ambulatory, you can have a quick puff on it now and hopefully you’ll find that helpful. So that was quite good that we could actually say you can have a vape”* [Advisor 01-06]

It was more difficult to gain medical staff buy-in at sites where vaping policies aligned with smoking policies (ie, being banned on NHS premises).

## Discussion

This is the first process evaluation study of a smoking cessation intervention evaluation in a UK ED. Findings demonstrated that despite contextual and implementation differences observed across sites as part of the COSTED trial, process evaluation data collected from participants, advisors, clinical staff, and researcher observations demonstrated that differences did not impact the outcome overall of smoking cessation.

Participant, staff, and observational data revealed contextual influences to act primarily as facilitators to intervention delivery, which have been shown to operate at the micro, meso, and macro levels of influence, despite what may be assumed about the busy ED environment being unsuitable for delivery of a smoking cessation intervention. Similar concerns have been expressed in other clinical contexts,^17,18^ where studies have similarly found that barriers are linked to staff capacity and training, resources, and norms, rather than participant acceptability concerns or the suitability of the context itself. In fact, participants often reported that the ED context was facilitative—the long wait times meant that there was time to receive the intervention and that its focus on improving health outcomes was brought more sharply into focus for participants by the acute health condition (a “teachable moment”). Furthermore, having the intervention delivered by a stop-smoking advisor who was perceived to be a member of staff within the ED was actively reassuring, legitimizing trying vaping for smoking cessation for some people who perhaps had been cautious. Similarly, where environmental prompts were clear, such as “no-smoking” but “vape friendly” signage supported by site policies, the environment was conducive to supporting smoking cessation by switching to vaping. Advisors demonstrated flexibility in delivering the intervention across varying times, places, and adapting the intervention manual to tailor to patient presenting factors. Advisors came from varied professional backgrounds, demonstrating that the intervention could be delivered by a range of trained advisors, with or without prior smoking cessation expertise. This suggests that different staffing models would be feasible for implementing this intervention more widely but having dedicated Advisors to focus on delivering the intervention was critical, as clearly ED clinical staff would not have the capacity to focus on intervention delivery alongside existing clinical roles.

To successfully implement the COSTED intervention consideration of contextual influences is important. Crucially, policies supportive of vaping and environmental cues such as signage were seen as facilitative. Although having space for delivery of the intervention both physically and within patient care pathways might be optimal, what appears to be more important is advisor flexibility and adaptability to the changing busy context of the ED. Clearly, patients are accepting of the challenges and limitations. Advisor sensitivity to these challenges appeared to mitigate any negative impact, ensuring that the intervention could be delivered either in private or semipublic, and at different points as necessary in the patient care pathway. These flexible adaptations were fundamental to ensuring successful intervention delivery, which appeared to play out in the remarkably similar rates of cessation observed across sites. Although the trial itself was not statistically powered to address contextual variation, the qualitative process evaluation data clearly demonstrates the adaptability of the intervention and the way in which context could be moderated.

This study is a critical contribution to understanding how the ED context may have influenced observed outcomes. A limitation of this study is that data were drawn from a selected qualitative sample and are not necessarily representative. Specifically, we were not able to gather the views of those not consenting to a further qualitative interview, who may overly represent those for whom the intervention did not work well. However, triangulation of data sources, combining site observations with interviews with participants and staff, has enabled an explanatory context for the trial outcome. Analysis of qualitative data was undertaken independently, with PPI input to facilitate interpretation.

The ED context was shown to clearly impact intervention delivery and receipt of smoking cessation interventions “in the moment.” Although context may be a barrier to intervention implementation, flexibility and adaptiveness of staff, and acceptability by participants, were critically facilitative factors. The time, place, and “moment” were powerfully experienced by participants who were amenable to the long-term behavior change of smoking cessation. This suggests that the ED is an opportune location to support smoking cessation to improve long-term health.

## Supplementary material

Supplementary material is available at *Nicotine and Tobacco Research* online.

ntae223_suppl_Supplementary_Appendix_S1

## Data Availability

Qualitative data is available on request to the corresponding author.
