# Supplementary material for: The Context of the Emergency Department as a Location for a Smoking Cessation Intervention—Process Evaluation Findings From the Cessation of Smoking Trial in the Emergency Department Trial
Source: Nicotine Tob Res. 2024 Nov 7;27(5):909–16. doi: 10.1093/ntr/ntae223 (PMC12012237; doi:10.1093/ntr/ntae223)
Supplement: ntae223_suppl_Supplementary_Appendix_S1 [file ntae223_suppl_supplementary_appendix_s1.docx]

**Appendix 1: COSTED Trial ED contexts**

| Site | Mean deprivation decile of participants | Setting | Staff | Smoking / vaping policies | Observed smoking / vaping behaviours | Intervention delivery |
| --- | --- | --- | --- | --- | --- | --- |
| 01 | 5.2 (2.7) | Busy ED in modern hospital outside of rurally located city | Some crossover between research and clinical roles | ‘No smoking’ signs outside department and on hospital site. 2 signs saying ‘no smoking or use of e-cigarettes’. Hospital policy was to permit vaping on hospital grounds outside | No observed smoking behaviour but visible smoking debris around all no smoking sign areas. 1 person observed to be vaping outside. | In small room specifically assigned for COSTED intervention delivery, or in other private spaces |
| 02 | 3.2 (1.) | Oppressive ED environment in old building. No windows. Busy and confusing layout | Research office located in the ED. All advisors were current or ex-ED staff | ‘No smoking’ signs on hospital doors. No vaping signage observed. No vaping policy as well as no smoking. | Lots of smoking debris observed. people actively smoking in hospital walkway area. Vaping also observed. | Interventions usually delivered in private room |
| 03 | 2.8 (1.4) | Compact ED, approx. 35 seats. In large residential area | Advisor separate from clinical staff. limited interaction | Faded no smoking signage outside. Clean and ‘green’ site with lots of planting. Hardly any smoking debris observed. Vaping allowed on site but signage to indicate this small. | No smoking or vaping observe don site. Smoking observed in neighbouring park (staff) | Intervention usually delivered in main waiting area. |
| 04 | 4.4 (2.8) | Large modern ED department as part of hospital which has a range of buildings of different ages. Modern calm waiting area, although large, approx. 100 seats. Clear signage in ED to explain patient journey | Research office separate from ED. Staff did not seem to interact much. | Smoking allowed on site in specified areas. Vaping is allowed lots of no smoking signage. Specific sign asking people not to smoke near entrance, to protect others. Vape friendly signage from SSS. | No smoking observed on site, in designated areas. But both smoking and vaping observed near ED entrance. Lots of tobacco debris though near ED entrance | Site for intervention delivery varied, but observed to take place in main waiting area of ED |
| Site 5 | 4.4 (2.7) | Large modern hospital on outskirts of major city. Large waiting room. Very busy, long wait times reported. Complex patient journey through department | Separate research team based in ED | ‘No smoking’ signs at entrance. No vaping signage observed. Staff unsure of vaping policy of hospital. | No smoking observed but tobacco litter to side of main entrance and by entrance doors | Intervention in public space in ED |
| Site 6 | 6.5 (2.1) | Large 1960s built hospital on outskirts of city | Adviser had developed good relationships with staff | ‘No smoking’ signage outside. Policy is no smoking or vaping on site | Very little tobacco debris outside | Intervention usually delivered in a semi-private consultation room |
